# Quality of Life of Patients With Chronic Kidney Disease Under Maintenance Hemodialysis and Their Caregivers: A Cross-Sectional Study

**DOI:** 10.7759/cureus.46651

**Published:** 2023-10-07

**Authors:** Ram Chandra Panthi, Milan Dhungana, Dipesh Poudel, Kushal Raj Joshi, Anupam Bista, Gyan Krishna Kayastha

**Affiliations:** 1 Internal Medicine, Patan Academy of Health Sciences, Lalitpur, NPL; 2 Internal Medicine, Universal College of Medical Sciences, Bhairahawa, NPL; 3 Internal Medicine, Nepal Armed Police Force (APF) Hospital, Kathmandu, NPL

**Keywords:** sf-36 score, quality of life, maintenance hemodialysis, chronic kidney disease, caregiver

## Abstract

Background: Maintenance hemodialysis (MHD) prolongs the life of patients with end-stage chronic kidney disease (CKD), but this process can change their lifestyle, affecting their quality of life (QoL). Patients with MHD require their caregivers' assistance in daily management and repeated hospital visitation. This places a burden on caregivers affecting their QoL. Both patient and caregiver form a unit during the caregiving process. This study aims to compare and correlate the QoL of patients with CKD under MHD with their caregivers, considering their common familial and socioeconomic backgrounds.

Methodology: This is a cross-sectional, comparative study in the Hemodialysis Unit of Patan Academy of Health Sciences (PAHS), Lalitpur, Nepal. Patients aged >14 years with CKD under MHD and caregivers staying with the patient at their resident place for a minimum of two months were included in the study. QoL of patients with CKD under MHD was compared with caregivers under different domains of the physical component summary (PCS) and mental component summary (MCS) scores using an SF-36 (Short form-36) health survey questionnaire. Data was collected and entered in Microsoft Excel 2010/Epi info version 7.2 and analyzed.

Results: The overall QoL of caregivers was better than CKD patients under MHD in terms of both PCS score (48.13 vs. 35.36) and MCS score (48.11 vs. 43.25) and was statistically significant (p-value: <0.001) in both scores. The patient’s QoL was not significantly correlated with the caregiver's PCS score (p-value: 0.635). Still, there was a significant correlation between QoL and MCS scores (p-value: 0.006). Similarly, caregivers had better QoL than CKD patients under MHD under all eight domains, which was statistically significant. No significant correlation was found between the frequency and duration of MHD with PCS and MCS scores of both patient and caregiver.

Conclusion: Overall, the physical and mental QoL of the caregiver was better than CKD patients under MHD. Further studies need to be conducted to assess the QoL of both groups compared to the healthy population to address the issue of hemodialysis patients and their caregivers.

## Introduction

Chronic kidney disease (CKD) patients in end-stage require lifelong maintenance hemodialysis (MHD) or a kidney transplant to survive. The global estimated prevalence of CKD is 13.4%, and those needing renal replacement therapy are between 4.9 and 7.08 million [1]. An estimated 434.3 million people have CKD in Asia, among which 2.9 million people need dialysis, and this population is projected to grow in the coming years [2].

Patient and caregiver quality of life (QoL) is affected after dependency on MHD. Caregivers are involved in helping patients to manage their chronic disease [3]. This role can affect them physically and mentally, so they can be considered "hidden patients" [4]. Since patients and caregivers form a unit during the caregiving process, it is essential to consider both patients and their caregivers' issues [5].

Although many studies have assessed QoL in patients of CKD under MHD and caregivers alone or in comparison with the average population, limited studies are comparing and correlating QoL between them. To date, no studies in Nepal have directly compared and correlated the QoL of hemodialysis patients directly with their caregivers. This study aimed to compare and correlate physical and mental aspects of QoL of CKD patients under MHD with their caregiver considering their similar familial and socioeconomic backgrounds and the significant amount of time given by the caregiver in the caregiving process.

## Materials and methods

Study design

This is a single-centered, cross-sectional, observational, comparative study done in the Hemodialysis Unit of Patan Academy of Health Sciences for a duration of one year from January 2021 to December 2021.

Participant characteristics

Patients aged >14 years undergoing MHD and caregivers staying together with the patient at his/her residential place for a minimum of two months were included; Whereas patients with other stages of CKD but not under MHD; patients or caregivers with other underlying chronic conditions like chronic obstructive lung disease, decompensated chronic liver disease, and underlying malignancy; and caregivers less than sixteen years of age were excluded. Patients or caregivers not willing to participate in the study were also excluded from the study.

Ethics approval and consent to participate

This study commenced after ethical approval from the institute's Institutional Review Committee (IRC) -PAHS (PMM2012311474). Informed consent was taken from participants before enrollment. Participants could withdraw from the study at any time without giving any reason during the study period. A statement indicating that the participant had understood all the information in the consent form and was willing to participate voluntarily was obtained.

Data collection

Data was collected from the patients and their caregivers from the Hemodialysis Unit to assess their QoL by using a short form-36 (SF-36) health survey questionnaire [6]. There were a total of 36 questions under eight domains: physical functioning (PF), role physical (RP), bodily pain (BP), general health (GH), vitality (VT), social functioning (SF), role emotional (RE), and mental health (MH). Each domain was transferred into a 0 to 100 scale because each question carried equal weight. It was then divided into two summary scores: physical component summary (PCS) score: PF, RP, BP, and GH have the higher weights for the PCS; and mental component summary (MCS) score: VT, SF, RE, and MH have higher weights for the MCS. Scoring for each question and SF-36 scores for PCS and MCS were calculated as per standard SF-36 Physical and Mental Health Scale guidelines [7].

Sampling and statistical analysis

The sample size was calculated by taking reference of variables of a similar study by Nagasawa H et al. with a confidence interval of 95% and power of 80% with a ratio of the sample size of two groups of study being 1:1 [6]. Taking consideration of PCS and MCS scores, the sample size was 51 and 39 in each group, respectively, and hence, the minimum sample size was 51 in each group (total: 102). We took a sample size of 88 in each group during our study. Data was entered in Microsoft Excel 2010/Epi info version 7.2 and analyzed. Statistical analysis was done after testing the normal distribution of data. The normality of data was tested by comparing a histogram of the sample data to a normal probability curve and using Kolmogorov-Smirnov (K-S) test. Since all the data had a non-normal distribution, the chi-square test for categorical variables, the Mann-Whitney test for continuous variables, and the Spearman correlation coefficient were applied for correlation. A p-value of less than 0.05 was considered significant.

## Results

The census method of data collection was used in this study. At the start of the study, there were 109 CKD patients undergoing MHD in PAHS. Of these, 21 patients were excluded as they met the exclusion criteria. So, 88 patients fulfilled the inclusion criteria, comprising 176 participants. (88 in each: CKD patients under the MHD and caregiver groups). The median age of CKD patients under MHD and caregiver was 48 and 41 years, respectively (Table 1). The overall QoL of caregivers was better than CKD patients under MHD regarding both PCS and MCS scores and was statistically significant (p<0.001) in both scores (Table 2).

**Table 1:** Baseline characteristics: median age and gender of CKD patients under MHD and caregiver MHD, maintenance hemodialysis; CKD, chronic kidney disease

Parameter	CKD patient under MHD (N=88)	Caregiver (N=88)	p-value
Age (in years)	48 (31.25, 60)	41 (21,50.75)	0.07
Gender (male %)	46 (52.3%)	39 (42.5%)	0.291

**Table 2:** Comparison of QoL between CKD patients under MHD and caregivers in terms of PCS and MCS scores MHD, maintenance hemodialysis; CKD, chronic kidney disease; PCS, physical component summary; MCS, mental component summary

SF-36 summary score	CKD patient under MHD (N=88), Median (Q1, Q3)	Caregiver (N=88), Median (Q1, Q3)	p-value
PCS score	35.36 (31.13, 41.7)	48.13 (41.39, 55.07)	<0.001
MCS score	43.25 (37.58, 49.44)	48.11 (41.16, 52.12)	<0.001

**Figure 1:**
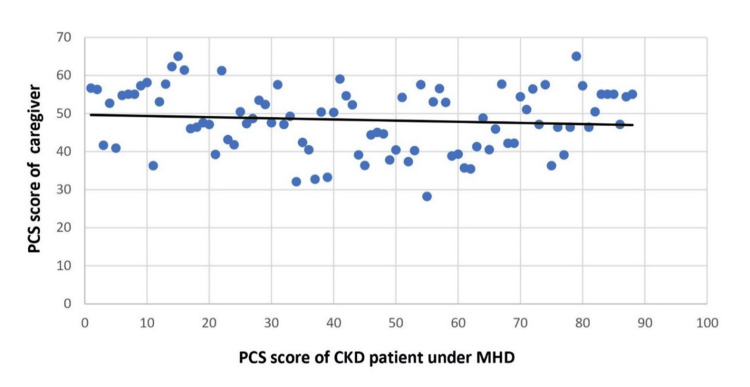
Correlation of PCS score between CKD patients under MHD and caregivers (spearman’s rho=0.051, p=0.634) MHD, maintenance hemodialysis; CKD, chronic kidney disease; PCS, physical component summary

**Figure 2:**
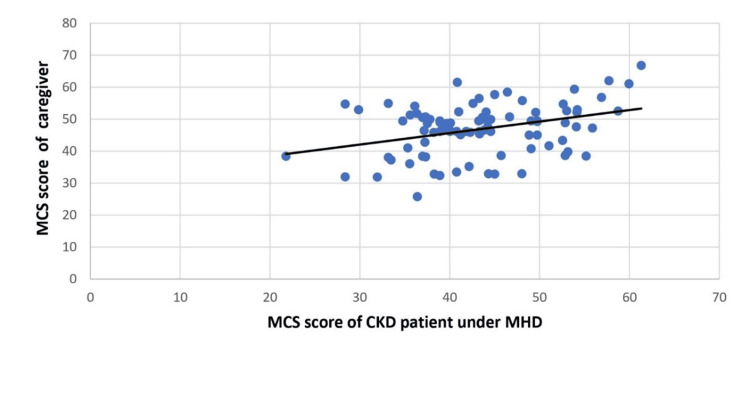
Correlation between MCS scores of CKD patients under MHD with caregivers (spearman’s rho=0.289, p=0.006) MHD, maintenance hemodialysis; CKD, chronic kidney disease; MCS, mental component summary

Patients' QoL was not significantly correlated with caregivers' QoL in terms of PCS score (p=0.635), but there was a significant correlation between QoL in terms of MCS score (p=0.006) (Figure 1 and Figure 2).

Similarly, caregivers also had better QoL than CKD patients under MHD under all eight PCS subdomains and MCS, which was statistically significant (Table 3). CKD patients had the lowest QoL under the subdomain RP and the lowest MCS under the subdomain RE. Caregivers had the lowest PCS under the subdomain GH and MCS under the subdomain RE.

**Table 3:** Comparison of QoL between CKD patients under MHD and caregivers in terms of PCS and MCS scores MHD, maintenance hemodialysis; CKD, chronic kidney disease; PCS, physical component summary; MCS, mental component summary; QoL, quality of life

SF-36 subdomains	CKD patient under MHD, N=88, Median (Q1, Q3)	Caregiver, N=88, Median (Q1, Q3)	p-value
PCS			
Physical functioning (PF)	50 (25,60)	82.5 (50,100)	<0.001
Role physical (RP)	25 (0,50)	75 (50,100)	<0.001
Bodily pain (BP)	67.5 (45, 87.6)	77.5 (67.5,90)	0.005
General health (GH)	40 (26.25, 50)	63.75 (55,80)	<0.001
MCS			
Vitality (VT)	50 (40,60)	69 (55,75)	<0.001
Role emotions (RE)	33.3 (0, 66)	66.6 (33.3,100)	0.005
Social functioning (SF)	75 (50,87.5)	75 (71.25, 100)	0.02
Mental health (MH)	60 (52,67.3)	68 (57,76)	<0.001

There was no significant correlation in the PCS score (p=0.72) and MCS score (p=0.13) of CKD patients under MHD with the frequency of hemodialysis in a week. Similarly, there was no significant correlation in the PCS score (p=0.13) and MCS score (p=0.64) of caregivers with the frequency of hemodialysis in a week (Table 4).

**Table 4:** Correlation of frequency of hemodialysis (in a week) with PCS and MCS scores of CKD patients under MHD and their caregivers MHD, maintenance hemodialysis; CKD, chronic kidney disease; PCS, physical component summary; MCS, mental component summary

	Frequency of hemodialysis	Once a week (N=3), Median (Q1)	Twice a week (N=60), Median (Q1, Q3)	Thrice a week (N=25), Median (Q1, Q3)	p-value
CKD patients under MHD	PCS score	32.3 (30.8)	35.95 (31.18, 42.18)	35.23 (30.54, 38.39)	0.72
	MCS score	37.24 (36.1)	42.93 (37.43,49.44)	43.32 (40.76,51.15)	0.13
Caregivers	PCS score	46.02 (32.7)	49.6 (41.9, 56.52)	46.41 (40.47,52.88)	0.13
	MCS score	48.63 (42.76)	48.18 (42.49,52.40)	46.1 (40.26,51.71)	0.64

**Figure 3:**
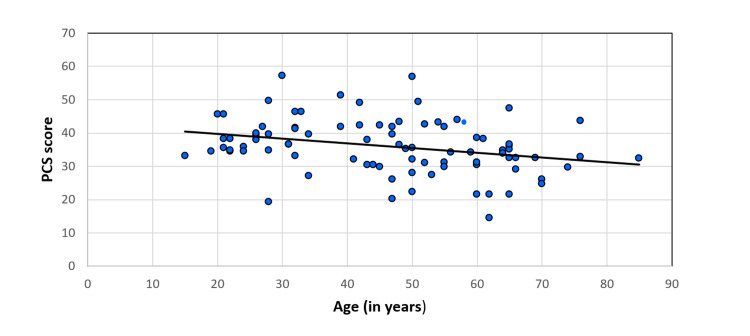
Correlation of age (in years) of CKD patients under MHD with PCS score (spearman rho: -0.33, p-value: 0.02) MHD, maintenance hemodialysis; CKD, chronic kidney disease; PCS, physical component summary

**Figure 4:**
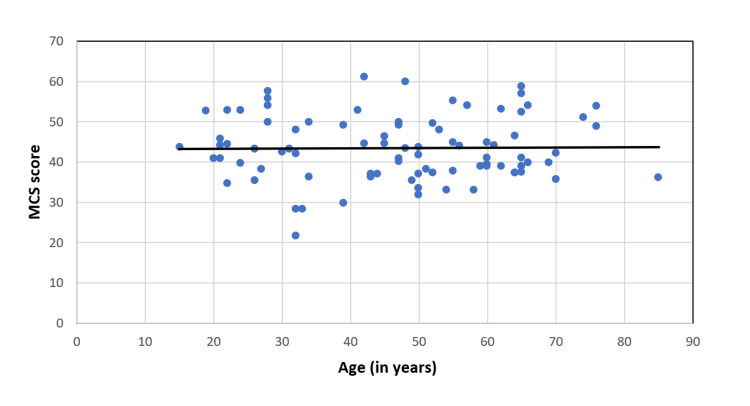
Correlation of age (in years) of CKD patients under MHD with MCS Score (spearman rho: -0.02, p-value: 0.84) MHD, maintenance hemodialysis; CKD, chronic kidney disease; MCS, mental component summary

The physical QoL of CKD patients under MHD decreased with age (spearman rho=-0.33) and was significant (p=0.02) (Figure 3). However, mental QoL was not significantly correlated with the advancing age of CKD patients under MHD (spearman rho: -0.02, p-value: 0.84) (Figure 4).

**Figure 5:**
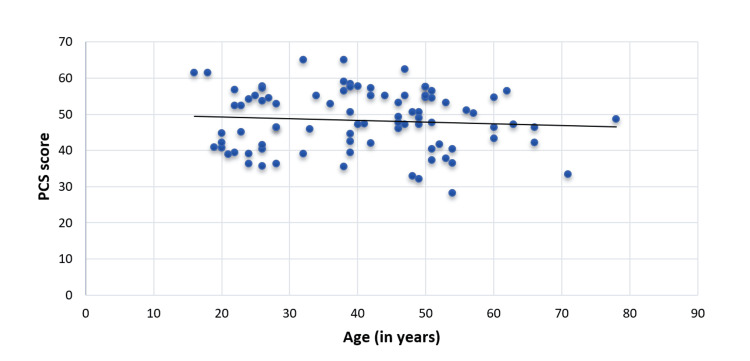
Correlation of age (in years) of caregivers with PCS score (spearman rho: -0.06, p-value: 0.57) MHD, maintenance hemodialysis; CKD, chronic kidney disease; PCS, physical component summary

**Figure 6:**
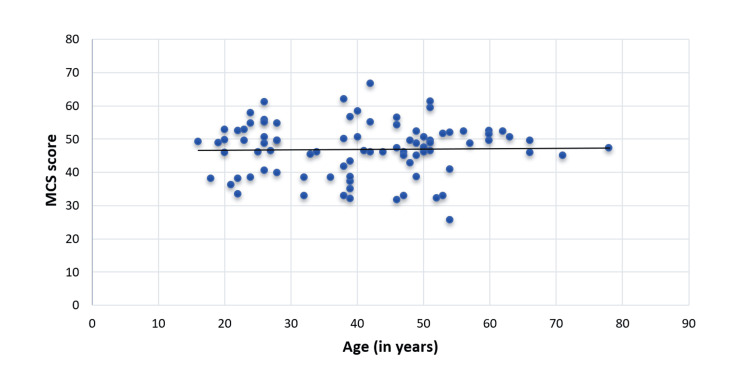
Correlation of age (in years) of caregivers with MCS score (spearman rho: -0.07, p-value: 0.53) MHD, maintenance hemodialysis; CKD, chronic kidney disease; MCS, mental component summary

There was no significant correlation between PCS and caregivers' MCS scores (Spearman rho: -0.06, p-value: 0.57) and advancing age (spearman rho: -0.07, p-value: 0.53) (Figure 5 and Figure 6).

The duration of MHD ranged from two months to 128 months, with the median duration being 18 months. No significant correlation was found between the duration of hemodialysis (in months) with PCS score (p-value: 0.12) and MCS score (p-value: 0.59) of CKD patients under MHD. Similarly, both PCS and MCS scores of caregivers were also not significantly correlated with the duration of hemodialysis (p=0.59 and p=0.25, respectively).

## Discussion

This study primarily compared the QoL of CKD patients under MHD and their respective caregivers. This study found that overall physical QoL as measured by PCS score was lower than that of their caregiver and was statistically significant. Caregivers also had statistically significantly better mental QoL than that patient. These findings differed from a similar study done by Gray NA et al. in which dialysis patients had poorer QoL but equivalent mental QoL compared to caregivers [7]. Poor QoL of hemodialysis patients might be attributed to the heavy burden associated with underlying kidney disease, as seen in the study by Nagasawa H et al. [8]. A study by Ehladad AA et al. showed poor mental QoL of end-stage kidney disease (ESKD) patients that could be attributed to multiple psychological stressors they face, such as anxiety, financial problems, difficulty in holding a job, fear of dying, and stress of underlying disease [9].

The patient's overall PCS score was less than MCS, like the findings in other studies (Gray NA et al., Nagasawa H et al., and Anu VK et al.) [7-8,10]. The deficit that existed for PCS score than MCS score was likely due to restrictions imposed by hemodialysis on the lives of these patients in their ability to participate in normal daily activities. The lowest score in hemodialysis patients under the PCS subdomain was in RP. A qualitative study by Roberti et al. showed that hemodialysis patients were limited by the symptoms of their disease and hemodialysis [11]. Dietary and fluid restrictions, the nature of hemodialysis treatment, toxin accumulation and uremic syndrome, fluid overload, and metabolic disorders contributed to the loss of energy in hemodialysis patients and were recognized as common physical complications in these patients. This study showed that the patient's most affected subdomain of MCS was RE, like Farzi S et al. [12]. Such emotional issues are likely due to anxiety, dependency on dialysis, and the feeling of being a burden on others as part of their condition. The least affected subdomain of the MCS of the patient was found to be SF. A similar finding was found by Anu VK et al., likely attributed to the solid familial support culture in Nepal regarding social interaction [10]. Family members are directly affected by the whole family and are committed to each other. This traditional structure is an essential source of support for the patient [13].

Caregivers had the highest score in PF under subdomains of PCS. This may be because, in our study, most caregivers were young to middle age group without any comorbidities. The lowest scores under PCS and MCS subdomains of caregivers were under GH and RE, respectively, as in other studies like Farzi S et al. and Nagasawa H et al. [9,12]. This may be because caregivers experience exhaustion, stress, and anxiety during the caregiving process.

In this study, the MCS score of patients was significantly correlated with that of caregivers, although there was no significant correlation between these two groups regarding PCS score. This might be because hemodialysis patients are the ones who must directly take the physical burden of underlying kidney disease and its treatment process. When a family member becomes ill, the whole family has an emotional concern. The study by Ibrahim N et al. showed a high correlation between the psychological well-being of caregivers and patients [14]. Gerogianni et al. study stated that caregivers have higher levels of anxiety and depression when dialysis patients under their care have high levels of anxiety and depression [15]. The physical QoL of life decreased with age and was significant (p-value: 0.02). These findings were consistent with other studies like Ishiwatari A et al., Van Loon IN et al., and Filipcic et al. [16-18]. However, mental QoL was not significantly correlated with the advancing age of CKD Patients under MHD (p-value: 0.68). These observations suggested that older patients are more restricted in their physical function than mental function, which was compatible with data from the worldwide DOPPS (Dialysis Outcomes and Practice Patterns Study) reported in 2011 [19]. Usually, the patients could adapt psychologically to their situation over time but not physically. With aging, there is a gradual and progressive reduction in their function capacity. This may limit their activity and daily living and consequently present worse QoL for dimensions associated with physical health [20]. Acceptance of the disease is essential in adapting to a chronic illness.

Caregivers did not have significantly decreased QoL in overall PCS and MCS scores with age. Caregiving may promote the maintenance of physical function through regular physical activity as part of daily care activities and may provide positive psychological benefits [21]. Also, their greater involvement in caregiving may have given them more satisfaction, resulting in health benefits. Caregivers may stay active by performing caregiving tasks or maintaining their health to continue assisting their care recipient. The provision of more frequent care is more representative of better health of the caregiver rather than the stress of the caregiving situation [22].

Our study did not show a significant correlation of QoL in the patient's physical and mental dimensions with the duration of hemodialysis. A survey by Morsch CM et al. and Barzegar et al. also had similar findings [23-24]. However, this contrasted with findings in other studies by Sethi et al. and Anees M et al. [25-26]. An increase in the duration of hemodialysis might cause the patients to adapt to hemodialysis and improve uremic symptoms to enhance their QoL. Similarly, no statistically significant correlation was found with QoL of patients with frequency of hemodialysis per week. Similar findings were found by Sethi et al. and Anees M et al., in which dialysis-related factor affecting QoL was evaluated [25-26]. However, in the study by Al Salmi I et al., physical QoL was affected by the increased frequency of dialysis [27]. Although increased frequency means an increased number of visits and more time spent in the hemodialysis center, there is improvement in uremic symptoms, fluid, and electrolyte balance with increased sessions of hemodialysis, which might have contributed to the better well-being of the patient.

In this study, caregivers' QoL was not significantly correlated to the frequency and duration of hemodialysis in terms of physical and mental dimensions. The study by Nataatmadja M et al. did not show a significant difference in SF-36 PCS and MCS scores of caregivers where comparison was made in caregivers of two groups that received hemodialysis 12 hours weekly to 24 hours weekly [28]. Although chronic medical conditions put many demands on caregivers, they adapt flexibly by gathering resources [29]. This may be because they likely get accustomed to the patient's dialysis treatment and daily care needs as the treatment period increases. 

There were a few limitations to this study. This was an observational cross-sectional study done in a single center. Other factors such as education level and occupation also could have affected the QoL of both patients and caregivers, which were not taken in this study. Although most of the studies we had compared with our study for assessing QoL used SF-36 as a generic questionnaire tool, some studies used different questionnaires, which might have resulted in some variation in interpreting the comparison of physical and mental QoL. Caregivers currently staying with the patient at their resident place were included in our study. However, the same caregiver might not have stayed with the patient for the total duration of the period for which the patient was under MHD. This might have an impact on the QoL scores of caregivers.

## Conclusions

Overall, caregivers' physical and mental QoL was better than CKD patients under MHD. QoL comparison was done between dialysis patients and caregivers, but there was no comparison with the normal population. Further studies need to be conducted to assess the QoL of both groups compared to the healthy population to address the QoL-related issue of hemodialysis patients and the caregivers who have vital roles in the caregiving process.
